# Long-Term Impact of Earthquakes on Sleep Quality

**DOI:** 10.1371/journal.pone.0055936

**Published:** 2013-02-13

**Authors:** Daniela Tempesta, Giuseppe Curcio, Luigi De Gennaro, Michele Ferrara

**Affiliations:** 1 Department of Life, Health and Environmental Sciences, University of L’Aquila, L’Aquila, Italy; 2 IRCCS S. Raffaele, Roma - Casa di Cura S. Raffaele, Cassino (FR), Italy; 3 Department of Psychology, “La Sapienza” University of Rome, Rome, Italy; Hôpital du Sacré-Coeur de Montréal, Canada

## Abstract

**Purpose:**

We investigated the impact of the 6.3 magnitude 2009 L’Aquila (Italy) earthquake on standardized self-report measures of sleep quality (Pittsburgh Sleep Quality Index, PSQI) and frequency of disruptive nocturnal behaviours (Pittsburgh Sleep Quality Index-Addendum, PSQI-A) two years after the natural disaster.

**Methods:**

Self-reported sleep quality was assessed in 665 L’Aquila citizens exposed to the earthquake compared with a different sample (n = 754) of L'Aquila citizens tested 24 months before the earthquake. In addition, sleep quality and disruptive nocturnal behaviours (DNB) of people exposed to the traumatic experience were compared with people that in the same period lived in different areas ranging between 40 and 115 km from the earthquake epicenter (n = 3574).

**Results:**

The comparison between L’Aquila citizens before and after the earthquake showed a significant deterioration of sleep quality after the exposure to the trauma. In addition, two years after the earthquake L'Aquila citizens showed the highest PSQI scores and the highest incidence of DNB compared to subjects living in the surroundings. Interestingly, above-the-threshold PSQI scores were found in the participants living within 70 km from the epicenter, while trauma-related DNBs were found in people living in a range of 40 km. Multiple regressions confirmed that proximity to the epicenter is predictive of sleep disturbances and DNB, also suggesting a possible mediating effect of depression on PSQI scores.

**Conclusions:**

The psychological effects of an earthquake may be much more pervasive and long-lasting of its building destruction, lasting for years and involving a much larger population. A reduced sleep quality and an increased frequency of DNB after two years may be a risk factor for the development of depression and posttraumatic stress disorder.

## Introduction

On April 6, 2009 at 3.32 a.m., an earthquake (6.3 Mw) hit the city of L'Aquila (Abruzzo), in central Italy, a town with 72,000 inhabitants and a health district of 105,000 residents. The earthquake caused the death of 309 people, with more than 1,600 individuals injured among whom 200 were severely injured and hospitalized, and 66,000 displaced.

Natural disasters like earthquakes are traumatic events that cause severe psychological distress. As a consequence, an increased risk for the development of posttraumatic stress disorder (PTSD), anxiety and depression has been consistently reported [1], [2], [3], [4], [5]. An increase of sleep complaints immediately after a trauma is also well documented [6], [7], [8]. In fact, altered sleep is a common and central symptom of PTSD. The most frequent self-related complaints (not always confirmed by polysomnographic recordings, for a review see [9]) are difficulties in falling asleep, frequent awakenings from sleep (with further difficulties falling back to sleep), shorter sleep duration, restless sleep, sleep-related breathing disorders (SRBD), daytime fatigue, nightmares and anxiety dreams.

Long-term evidence can be essential to establish the need of specific interventions for the prevention and treatment of mental disorders that may occur in the years after a traumatic experience. Only few longitudinal studies [10], [11], [12], [13], [14] have investigated the effects of extreme situational stress on sleep. After traumatic episodes of various types such as combat [10], [14], [15], [16], marine explosions [17], earthquakes [13], [18], [19] and car crashes [20], a high incidence of severe sleep disturbances has been shown. These effects can be long lasting. In the above mentioned follow-up studies it was found that traumatic experiences continued to show their effects on sleep from 8 months to 15 years after the trauma [10], [13], [17].

A significant lengthening of REM latency and a reduction of REM time has been also reported in postcombat subjects investigated in the sleep laboratory about 2 years after their traumatic experiences [11], [12]. However, in all the studies the samples were very small and not representative of the affected population. Most of these studies were based on polysomnographic data, that usually do not correlate with subjective complaints about sleep, in both normal and pathological individuals [21].

For these reasons, we decided to investigate the impact of the earthquake that occurred on April 6, 2009, in L'Aquila, on the subjective sleep quality of a large sample of the population living in the city and in the surroundings. Sleep quality was assessed by means of the Pittsburgh Sleep Quality Index (PSQI), one of the most frequently used self-report instruments to assess sleep quality in healthy and psychiatric samples [22], [23], [24], [25].

Moreover, in order to measure the presence and severity of trauma-related subjective sleep disturbances in the people who have been exposed to the earthquake, the Pittsburgh Sleep Quality Index-Addendum (PSQI-A; [26]) was administered. The PSQI-A is a more specific self-report measure that evaluates the frequency of seven disruptive nocturnal behaviours common among individuals affected by PTSD. These behaviours may adversely contribute to PTSD severity and outcomes over the time [27].

Therefore, we retrospectively investigated whether: a) subjective sleep quality of L'Aquila citizens changed two years after the earthquake compared to two years before it; b) two years after the earthquake, the self-reported sleep quality of this population differed from that of people living between 40 and 115 km from the epicenter.

## Methods

### Ethics Statement

The protocol was approved by the Ethics Review Commitee of the Faculty of Psychology and was conducted in accordance with the Declaration of Helsinki, with the written consent of each subject.

### Participants

**Figure 1:**
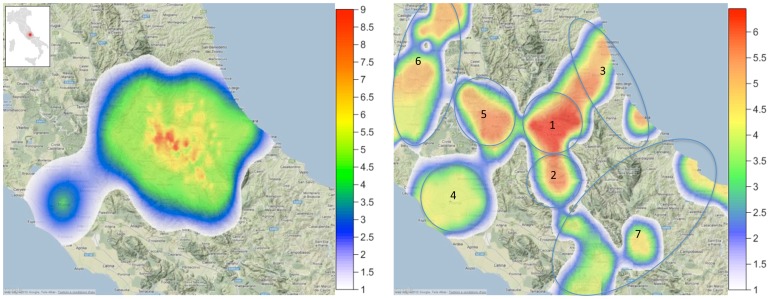
Seismic and sleep disturbance intensity. *Left Panel*: Plot of the seismic intensity of the earthquake who hit L'Aquila on 6 april 2009. The map covers a large portion of central Italy (as shown in red in the small inset map), from the Thyrrenian to the Adriatic sea. Data are plotted according to the European Macroseismic Scale (EMS-98), the basis for evaluation of seismic intensity in European countries (Grünthal, 1998). At variance with the earthquake magnitude scales, which express the seismic energy released by an earthquake, EMS-98 intensity denotes how strongly an earthquake affects a specific place. The EMS-98 has 12 divisions, from I (not felt) to XII (completely devastating). In this case, the maximal intensity reached was IX-X (IX = Destructive: monuments and columns fall or are twisted. Many ordinary buildings partially collapse and a few collapse completely; X = Very destructive: many ordinary buildings collapse). *Right Panel*: Plot of the mean colour-coded scores to the Pittsburgh Sleep Quality Index (PSQI) obtained two years after the earthquake by each of the seven groups numbered (within the ovals) as in Table 1. Higher PSQI scores (>5, red-orange colours) indicate the presence of clinically relevant sleep disturbances. The geographic area interested by this effect is appreciably broader than the area affected by the most destructive effects of the earthquake (*Left Panel*, red areas).

Before the proper experimental phase, the present study was advertised to several thousands people living in the Abruzzo region (in which the earthquake's epicenter was localized) and in neighboring regions (Lazio, Molise, Marche, Umbria). We selected geographic areas that were conveniently located (easy to reach or where the experimenters lived), with the aim of maximize the probability to having the questionnaires filled in. A total population of 5332 people were interested in the study and, after a balance of the potential sample for gender and age (range 20–80 years), such a group was contacted to participate in the study. Of these, 5163 (96.8%) actually agreed to participate and filled-in the questionnaires. The participants who did not complete the questionnaires in every part were excluded by the final analysis. As a consequence, a sample of 4993 individuals (96.7% of the total participants) was assigned to eight different groups (see Figure 1) and entered the analyses. For the analyses purposes, all the participants were subdivided into three separate age groups [young adults (20–40 years; n = 1931), adults (41–60 years; n = 1758), elderly (61–80 years; n = 1304)] and on the basis of gender (for more information, see Table 1).

**Table 1:** Geo-demographic characteristics of the sample.

GROUPS	Mean distance (Km) from the epicenter	Total participants	Gender	Age
Group 1-PRE (L’Aquila, Abruzzo)	0	754	F = 367	Young adults = 117 Adults = 162 Elderly = 88
			M = 387	Young adults = 131 Adults = 173 Elderly = 83
Group 1-POST (L’Aquila, Abruzzo)	0	665	F = 340	Young adults = 171 Adults = 99 Elderly = 70
			M = 325	Young adults = 154 Adults = 91 Elderly = 80
Group 2 (Avezzano, Teramo, Rieti: Abruzzo-Lazio)	40	739	F = 385	Young adults = 153 Adults = 132 Elderly = 100
			M = 354	Young adults = 150 Adults = 112 Elderly = 92
Group 3 (Pescara, San Benedetto: Abruzzo-Marche)	73 (eastward)	451	F = 231	Young adults = 91 Adults = 85 Elderly = 55
			M = 220	Young adults = 84 Adults = 70 Elderly = 66
Group 4 (Roma, Viterbo: Lazio)	99 (westward)	563	F = 279	Young adults = 106 Adults = 93 Elderly = 80
			M = 284	Young adults = 106 Adults = 97 Elderly = 81
Group 5 (Sora, Cassino, Formia: Lazio)	101 (northwestward)	686	F = 335	Young adults = 121 Adults = 119 Elderly = 95
			M = 351	Young adults = 131 Adults = 112 Elderly = 108
Group 6 (Perugia, Orvieto, Terni: Umbria-Lazio)	99 (southeastward)	649	F = 321	Young adults = 116 Adults = 125 Elderly = 80
			M = 328	Young adults = 122 Adults = 118 Elderly = 88
Group 7 (Vasto, Isernia, Fornelli, Termoli: Abruzzo-Molise)	115 (southeastward)	486	F = 236	Young adults = 84 Adults = 82 Elderly = 70
			M = 250	Young adults = 94 Adults = 88 Elderly = 68

The whole sample (n = 4993) was subdivided into seven different groups, based on the place of residence of the participants (cities and regions are also reported). The second column shows, for each group, the mean distance from the epicenter of the earthquake. In the other columns, the number of participants and the gender and age composition of each group are reported.

The first group (Group 1-PRE) includes a sample of L'Aquila citizens tested 24 months before the 2009 earthquake, for the purpose of another study investigating the relationship between sleep quality, alexithymia and some personality traits. These data have not been published before elsewhere. Since the 754 participants of the pre-earthquake cohort filled in the questionnaires anonymously, it was impossible to re-contact them. Therefore, the second group (Group 1-POST) includes 665 different L'Aquila citizens exposed to the 2009 earthquake.

Participants in the other six groups lived in different areas ranging between 40 and 115 km from the earthquake epicenter (Figure 1). These groups were created on a geopolitical basis. Starting from the Abruzzo region and moving toward the bordering regions, we grouped people living in proximal areas (within the same region or on the border of two regions) in order to have roughly equivalent group sizes.

All these groups were tested 24 months after the traumatic experience. For each group, place of residence of the participants (city and region), distance from the epicenter, size and composition are reported in Table 1.

### Assessments

The Pittsburgh Sleep Quality Index (PSQI), an instrument with previously established reliability and validity [22], [28] was adminstered to evaluate sleep quality before and after the earthquake in two different groups of L'Aquila citizens and after the earthquake in all the other groups. The PSQI assesses seven components of sleep quality (subjective sleep quality, sleep latency, duration, efficiency, disturbances, use of sleep medication, and daytime dysfunction). Each component is rated on a 0–3 severity scale referring to the frequency of each disturbance (0 = not in the past month, 1 = less than once a week, 2 = once or twice a week, 3 = three or more times a week) and yields a global score with a range of 0–21 [22]. A PSQI global score of ≥5 suggest clinically significant sleep disturbances in a healthy population.

Moreover, all the participants tested after the earthquake were also administered the PSQI-A [26] and the Beck Depression Inventory (BDI; [29]). The PSQI-A assesses the frequency, during the previous month, of seven disruptive nocturnal behaviours (DNB): hot flashes, general nervousness, memories or nightmares of traumatic experience, severe anxiety or panic not related to traumatic memories, bad dreams not related to traumatic memories, episodes of terror or screaming during sleep without fully awakening, episodes or acting out dreams, such as kicking, punching, running, or screaming. A PSQI-A score >4 is highly predictive for discriminating between subjects with and without PTSD [26].

The BDI is a multiple-choice self-report inventory assessing depressive symptoms. The cut-off score for depression is usually set above 11.

### Data Analyses

A factorial ANOVA Gender (Females vs. Males)×Age (Young Adults vs. Adults vs. Elderly)×Time (Pre-earthquake vs. Post-earthquake) was carried out on PSQI global score, to evaluate pre-post earthquake sleep quality changes in the two different L'Aquila samples, and the existence of potential moderating effects of age and gender.

To assess the impact of the earthquake on sleep quality of people living in L'Aquila and in the surroundings, a factorial ANCOVA design was used with BDI scores as a covariate. In fact preliminary analyses showed significant correlations between PSQI and BDI scores (r = 0.47; p = 0.000001) and PSQI-A and BDI scores (r = 0.31; p = 0.000001).

Thus, a factorial ANCOVA Group [1-POST vs. 2 vs. 3 vs. 4 vs. 5 vs. 6 vs. 7]×Gender [Females vs. Males]×Age [Young Adults vs. Adults vs. Elderly] was carried out on PSQI global score and PSQI-A.

For all the analyses, in case of significant effects, Scheffè *post-hoc* tests were carried out. The level of significance was always set at p<0.05.

Finally, to further evaluate the effects of proximity to the epicenter and of depression on sleep quality and DNBs in all groups evaluated *after* the earthquake, a multiple regression model was applied, using the above measures as independent variables and PSQI and PSQI-A global scores as dependent variables. Age and gender were also included as predictors, with the aim to partialling out their contribution.

## Results

### Pre-Post Earthquake Sleep Quality in the city of L’Aquila

Table 2 shows means (and SEM) of the sleep quality indexes (PSQI and PSQI-A) and of BDI scores for each group, as a function of gender and age. *Post hoc* comparisons of the significant interactions are also reported.

**Table 2:** Mean scores (±SEM) to the Pittsburgh Sleep Quality Index (PSQI), the Pittsburgh Sleep Quality Index-Addendum (PSQI-A) and Beck Depression Inventory (BDI) reported by the participants living in L'Aquila before (Group 1-PRE) and after the earthquake (Group 1-POST) and in the other geographic areas (Groups 2, 3, 4, 5, 6, 7), as a function of gender (Females, F; Males, M) and age (Young Adults, YA; Adults, A; Elderly, E).

	PSQI	PSQI-A	BDI	Post-hoc (PSQI)	Post-hoc (PSQI-A)
**Group 1-PRE**	**1)** 4.52 (0.09)	–		1 vs 7****	49 vs 61***
F	**2)** 4.78 (0.14)	–		6 vs 12****	49 vs 67***
M	**3)** 4.27 (0.13)	–		5 vs 11****	49 vs 73***
YA	**4)** 4.19 (0.13)	–		4 vs 10****	49 vs 79***
A	**5)** 4.43 (0.14)	–		7 vs 13****	49 vs 85***
E	**6)** 4.94 (0.19)	–		7 vs 19****	55 vs 61***
**Group 1-POST**	**7)** 6.33 (0.10)	**49)** 6.53 (0.16)	10.80 (0.26)	7 vs 25****	55 vs 67***
F	**8)** 6.37 (0.15)	**50)** 6.73 (0.23)	10.79 (0.37)	7 vs 31****	55 vs 73***
M	**9)** 6.30 (0.15)	**51)** 6.34 (0.23)	10.81 (0.37)	7 vs 37****	55 vs 79***
YA	**10)** 5.53 (0.14)	**52)** 5.86 (0.22)	9.36 (0.36)	7 vs 43****	55 vs 85***
A	**11)** 6.32 (0.18)	**53)** 6.42 (0.29)	9.82 (0.47)	13 vs 25****	59 vs 77***
E	**12)** 7.15 (0.21)	**54)** 7.32 (0.33)	13.22 (0.53)	13 vs 31****	60 vs 78***
**Group 2 (40 km)**	**13)** 5.59 (0.09)	**55)** 4.80 (0.15)	9.43 (0.24)	18 vs 16****	52 vs 76**
F	**14)** 5.90 (0.13)	**56)** 5.35 (0.21)	10.33 (0.33)	13 vs 43****	52 vs 82**
M	**15)** 5.28 (0.14)	**57)** 4.25 (0.22)	8.52 (0.35)	19 vs 25****	52 vs 88**
YA	**16)** 4.67 (0.14)	**58)** 4.80 (0.23)	7.26 (0.37)	19 vs 31****	52 vs 64**
A	**17)** 5.55 (0.16)	**59)** 4.85 (0.26)	8.84 (0.41)	17 vs 29***	52 vs 70**
E	**18)** 6.56 (0.18)	**60)** 4.75 (0.29)	12.18 (0.47)	17 vs 35***	53 vs 65**
**Group 3 (73 km eastward)**	**19)** 5.25 (0.12)	**61)** 3.64 (0.19)	7.57 (0.31)	11 vs 29***	53 vs 71**
F	**20)** 5.53 (0.17)	**62)** 4.29 (0.17)	8.55 (0.44)	11 vs 35***	53 vs 77**
M	**21)** 4.97 (0.17)	**63)** 2.98 (0.27)	6.59 (0.44)	11 vs 47***	53 vs 83**
YA	**22)** 4.60 (0.19)	**64)** 3.34 (0.31)	6.06 (0.49)	10 vs 28**	53 vs 89**
A	**23)** 4.86 (0.20)	**65)** 3.67 (0.33)	7.35 (0.52)	10 vs 34**	54 vs 60**
E	**24)** 6.30 (0.23)	**66)** 3.90 (0.37)	9.30 (0.59)	10 vs 46**	54 vs 66**
**Group 4 (99 km westward)**	**25)** 4.03 (0.10)	**67)** 3.05 (0.17)	5.19 (0.27)	12 vs 24**	54 vs 72**
F	**26)** 4.06 (0.15)	**68)** 3.44 (0.24)	5.62 (0.39)	12 vs 30**	54 vs 78**
M	**27)** 4.01 (0.15)	**69)** 2.65 (0.24)	4.77 (0.38)	12 vs 36**	54 vs 84**
YA	**28)** 3.88 (0.17)	**70)** 3.31 (0.28)	4.45 (0.44)	12 vs 48**	54 vs 90**
A	**29)** 3.92 (0.18)	**71)** 2.96 (0.29)	4.88 (0.47)	18 vs 30**	
E	**30)** 4.30 (0.20)	**72)** 2.87 (0.32)	6.25 (0.51)	18 vs 36**	
**Group 5 (101 km northwestward)**	**31)** 3.94 (0.09)	**73)** 2.57 (0.15)	7.26 (0.25)	18 vs 48**	
F	**32)** 3.91 (0.14)	**74)** 3.05 (0.22)	7.95 (0.25)	19 vs 43**	
M	**33)** 3.96 (0.13)	**75)** 2.08 (0.22)	6.57 (0.34)	13 vs 37*	
YA	**34)** 3.87 (0.16)	**76)** 3.33 (0.26)	5.57 (0.41)	24 vs 22*	
A	**35)** 3.90 (0.17)	**77)** 2.28 (0.27)	6.36 (0.42)	11 vs 23*	
E	**36)** 4.03 (0.18)	**78)** 2.09 (0.29)	9.85 (0.45)	12 vs 10°°	
**Group 6 (99 km southeastward)**	**37)** 5.02 (0.10)	**79)** 3.02 (0.16)	7.73 (0.26)	12 vs 10°	
F	**38)** 5.24 (0.14)	**80)** 3.41 (0.23)	8.62 (0.37)		
M	**39)** 4.79 (0.14)	**81)** 2.63 (0.23)	6.83 (0.36)		
YA	**40)** 4.55 (0.16)	**82)** 3.63 (0.26)	6.09 (0.42)		
A	**41)** 5.12 (0.16)	**83)** 2.92 (0.26)	8.00 (0.41)		
E	**42)** 5.38 (0.20)	**84)** 2.50 (0.31)	9.08 (0.50)		
**Group 7 (115 km southeastward)**	**43)** 4.45 (0.11)	**85)** 3.15 (0.18)	6.90 (0.29)		
F	**44)** 4.52 (0.16)	**86)** 3.75 (0.26)	7.22 (0.42)		
M	**45)** 4.38 (0.16)	**87)** 2.55 (0.26)	6.58 (0.41)		
YA	**46)** 4.02 (0.19)	**88)** 3.17 (0.30)	5.62 (0.49)		
A	**47)** 4.45 (0.19)	**89)** 3.07 (0.31)	6.67 (0.50)		
E	**48)** 4.86 (0.20)	**90)** 3.21 (0.35)	8.41 (0.55)		

Results of the significant *post-hoc* comparisons of the ANOVA and ANCOVA interactions are also shown (see text for further details).

**** = p<0.0001.

*** = p<0.001.

** = p<0.01.

* = p<0.05.

°° = p<0.0001, referred to the *post-hoc* comparison of the ANOVA Time×Age interaction.

° = p<0.01, referred to the *post-hoc* comparison of the ANCOVA Group×Age interaction.

After the earthquake, we found a statistically significant decline of sleep quality in the L'Aquila citizens (main effect for Time: F_1,1407_ = 156.16, p<0.0000001; pre-earthquake PSQI scores, mean±SEM: 4.52±0.09, post-earthquake: 6.33±0.10).

The main effects for Gender (F_1,1407_ = 3.94; p<0.05) and Age (F_2,1407_ = 21.26; p<0.0000001) were also significant.

**Figure 2:**
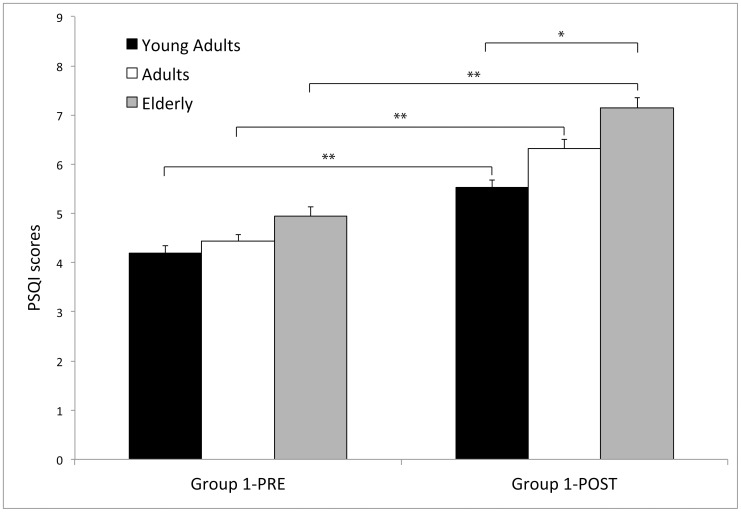
Mean (±SEM) Pittsburgh Sleep Quality Index (PSQI) global scores reported by the participants living in L'Aquila before (Group 1-PRE) and after the earthquake (Group 1-POST). Data are plotted as a function of the age (Young Adults, Adults and Elderly). Before the earthquake the three age groups did not differ between them. After the earthquake, the Elderly showed a worse sleep quality compared to the Young Adults (*p<0.0000001). All the age subgroups showed a deterioration of sleep quality after the earthquake (**p<0.000001).

The Time×Age interaction (F_2,1407_ = 3.10; p<0.05; see Figure 2) indicated that, before the traumatic experience, the three age groups did not differ (all probabilities were 0.94<p>0.11). Instead, after the earthquake the Elderly showed a worse sleep quality compared to the Young Adults (p<0.0000001), but not compared to the Adults group (p = 0.13). Moreover, sleep quality in the Adults was tendentially worse than in the Young Adults (p = 0.06).

All the age subgroups showed a deterioration of sleep quality after the earthquake compared to the pre-earthquake scores (all p<0.000001).

Finally, the remaining interactions were not significant (Time×Gender: F_1,1407_ = 2.40; p = 0.12; Age×Gender: F_2,1407_ = 0.19; p = 0.82; Time×Age×Gender: F_2,1407_ = 1.67; p = 0.18).

### Post-Earthquake Sleep Quality: PSQI Differences Between L’Aquila and the Surroundings

The ANCOVA Group×Gender×Age on PSQI scores showed a significant main effect for Group (F_6,4196_ = 40.09; p<0.0000001). After having partialled out the effect of the covariate (F_1,4196_ = 940.07; p<0.000001), sleep quality of L’Aquila citizens (Group 1, mean±SEM = 6.33±0.10) was largely worse than all the other groups (Groups 2–7; p<0.000001 for all comparisons). Further post-hoc analyses, limited to the other groups showing PSQI scores above the cutoff (mean±SEM: Group 2 = 5.59±0.09, Group 3 = 5.25±0.12), also showed that Group 2 reported a worse sleep quality compared to Group 4 (p<0.000001), 5 (p<0.000001), 6 (p<0.05) and 7 (p<0.000001). Moreover, Group 3 showed higher PSQI scores than Group 4 (p<0.000001), 5 (p<0.000001) and 7 (p<0.01).

The main effect for Age was significant (F_2,4196_ = 13.79; p<0.000001), while the main effect for Gender was not significant (F_1,4196_ = 0.56; p = 0.45).

**Figure 3:**
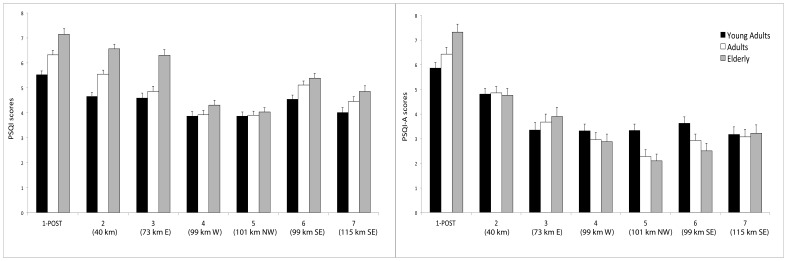
Mean scores (±SEM) to the Pittsburgh Sleep Quality Index (PSQI; *left panel*) and to the Pittsburgh Sleep Quality Index-Addendum (PSQI-A; *right panel*) for the seven geographic groups divided in three age subgroups (Young Adults, Adults and Elderly). Please see the Results section for more details.

The interaction Group×Age was also significant (F_12,4196_ = 4.17; p<0.00001). The Elderly showed poorer sleep quality than Young Adult subjects in Group 1 (p<0.005), 2 (p<0.000001) and 3 (p<0.05), as depicted in Figure 3 (left panel).

As far as between groups are concerned, we focused on the differences between Groups 1 and 2 (living in L'Aquila and the immediate surroundings) and all the other Groups (3–7). Young Adults living in L'Aquila (Group 1) showed higher PSQI scores than those of Groups 4, 5 and 7 (all p<0.001). L'Aquila Adults had higher scores than those of Groups 3 (p<0.05), 4, 5 and 7 (all p<0.0001). Group 1 Elderly participants reported lower sleep quality than those of Groups 3, 4, 5 and 7 (all p<0.001).

Moreover, Adults living within 40 km from the epicenter (Group 2) showed higher PSQI scores than those in Groups 4 and 5 (p<0.0001). Elderly participants of the same group reported higher scores than those in Groups 4, 5 and 7 (all p<0.001).

The Gender×Age interaction was also significant (F_2,4196_ = 4.73 p = 0.008) indicating a worse sleep quality in Elderly females compared to Young Adult males and females, Adult males and females and Elderly males (all p between 0.05 and 0.000001). Moreover, Elderly males showed higher PSQI scores than Young Adult and Adult females (p<0.001 and 0.00001).

All the remaining interactions were not significant (Group×Gender: F_6,4196_ = 1.18, p = 0.31; Group×Gender×Age: F_12,4196_ = 1.21, p = 0.26).

### Post-Earthquake Sleep Quality: Multiple Regressions

Although the above reported effects were obtained partialling out the contribution of depression, the effect of the covariate was nevertheless significant. Therefore, we carried out a multiple regression with distance from the epicenter, gender, age and depression scores as independent variables. As shown in Table 3, all the predictors but gender significantly entered in the regression equation.

**Table 3:** Upper section: Results of the multiple regressions considering PSQI global scores as dependent variable, and distance from the epicenter, gender, age and depression (BDI scores) as predictors.

Dependent variable: PSQI global scores, Multiple R = 0.50, F4,4234 = 355.99, p<0.00000001				
Indipendent Variables	β Coeff.	Partial Corr.	t(4234)	p
**Distance**	−0.15	−0.17	−11.28	0.0000001
**Gender**	−0.004	−0.004	−0.3	0.76
**Age**	0.07	0.08	5.16	0.000001
**BDI scores**	0.43	0.43	30.74	0.00000001
**Dependent variable PSQI-A global scores, Multiple R = 0.40, F4,4234 = 199.72, p<0.0000001**				
**Indipendent Variables**	**β Coeff.**	**Partial Corr.**	**t(4234)**	**p**
**Distance**	−0.21	−0.21	−14.6	0.00000001
**Gender**	−0.07	−0.08	−5.08	0.000001
**Age**	−0.07	−0.08	−5.12	0.000001
**BDI scores**	0.28	0.28	18.94	0.00000001

The table reports beta weights, partial correlation coefficients, t values and probability. Lower section: Results of the multiple regressions considering PSQI-Addendum global scores as dependent variable, and distance from the epicenter, gender, age and depression (BDI scores) as predictors.

Although depression scores were the best predictor, it is of note that the distance from the epicenter still significantly predicts PSQI scores, even partialling out the strong influence of depression. Finally, age was positively related to PSQI scores, confirming the well-known progressive decrease of sleep quality with age.

### Post Earthquake DNB: PSQI-A Differences between L’Aquila and the Surroundings

In keeping with the above results, partialling out the influence of the covariate (F_1,4196_ = 360.75; p<0.000001) the ANCOVA on PSQI-A scores showed a main effect for the Group (F_6,4196_ = 47.81; p<0.0000001). Post-hoc analyses, limited to the groups showing PSQI-A scores above the cutoff (mean±SEM: Group 1 = 6.53±0.16, Group 2 = 4.80±0.15), showed the highest incidence of disrupted nocturnal behaviors in L'Aquila citizens and in people living within 40 km from the epicenter (Groups 1 and 2) in comparison to all the other groups (Groups 3–7; p<0.0001 for all post hoc comparisons).

The main effects for Gender (F_1,4196_ = 32.78, p<0.000001) and Age (F_2,4196_ = 10.48 p<0.00005) were also significant.

The Group×Age interaction was significant (F_12,4196_ = 3.18; p<0.005). Although a higher incidence of DNB was apparently present at least in the elderly L’Aquila citizens (Figure 3, right panel), and a higher incidence of DNB was present in the Groups 4–6, post hoc comparisons did not show significant differences between different ages within any group.

As far as between groups are concerned, also in this case we focused on the differences between Groups 1 and 2 (living in L'Aquila and the immediate surroundings) and all the other Groups (3–7). Young Adults living in L'Aquila showed higher PSQI-A scores than those of Groups 3–7 (all p<0.001). Group 1 Adults reported higher scores than those of Groups 3–7 (all p<0.001). Group 1 Elderly showed higher PSQI-A scores than all the other groups (p<0.001).

In Group 2, Adults and Elderly participants differed from the corresponding age subgroups in Group 5 (p<0.0001 and p<0.0005, respectively).

The remaining interactions were not significant (Group×Gender: F_6,4196_ = 0.52, p = 0.78; Gender×Age: F_2,4196_ = 0.97, p = 0.37; Group×Gender×Age: F_12,4196_ = 1.74, p = 0.06).

### Post-Earthquake DNB: Multiple Regressions

Also in the case of DNB, although the above reported effects were obtained partialling out the contribution of depression, the effect of the covariate was significant. Therefore, as for PSQI global scores, we carried out the same multiple regressions with distance from the epicenter, gender, age and depression scores as independent variables. As shown in Table 3, all the independent variables entered the regression equation. It is of note that, in this case, the contribution of depression scores and of distance from the earthquake epicenter was similar.

Gender also entered the regression equation, indicating a prevalence of post-earthquake DNBs among women. Finally, age showed a negative relation with DNBs, suggesting that the specific effects of the earthquake on DNBs may be more evident in young than in elderly people.

## Discussion

Here we investigated the long-term effects of the L’Aquila earthquake (April 6, 2009) on subjective sleep quality and trauma-related disruptive nocturnal behaviors. We showed that, even after a period of two years, people exposed to a catastrophic disaster continue to suffer from a reduced sleep quality and an increased frequency of disruptive nocturnal behaviors.

More specifically, the comparison between L’Aquila citizens before and after the earthquake showed that such a traumatic experience significantly degraded sleep quality. Although this effect was present in all age groups, sleep quality deterioration was more pronounced in the elderly.

The comparison between people living closer to the earthquake epicenter with those living in farther cities evidenced an interesting effect on sleep quality. In fact, we showed an increase of PSQI scores as a function of the (shorter) distance from the epicenter, indicating a clear reduction of sleep quality in a range of about 70 km (Group 3) around L’Aquila. This effect was largely influenced by age. Also in this case the elderly appeared more vulnerable to the traumatic experience: in fact, older individuals living closer to the epicenter complained the worst sleep quality. Beyond the distance of about 70 km, participants tended to show normal scores of self-assessed sleep quality. This effect was independent by the possible influence of depression, since the effect of this variable was statistically partialled out.

The use of two different statistical approaches (covariation and multiple regression analyses) indeed suggests that distance from the epicenter and depression may have strong but also independent effects on sleep quality, as assessed by PSQI global scores. In particular, the first approach clearly showed a robust effect of the covariate, together with the persistence a significant effect of deterioration of sleep quality also after having partialled out the depression contribution. On the other hand, the regression approach showed that depression is the strongest predictor of PSQI global scores, but again the influence of proximity to the epicenter remains significant after having excluded the influence of depression. Therefore one may argue that the earthquake had a negative effect on both sleep quality and depression, and that only part of the deterioration of sleep quality can be related to the depressive mood. The multiple regression approach also indicated that post-earthquake sleep quality progressively decreases with age. This might point to a higher vulnerability of older people to develop sleep disturbances as a consequence of the exposure to a trauma. This result apparently contradicts those of one study reporting no association between age and sleep quality in a sample of PTSD patients, half of which reported psychiatic comorbidity - mood and/or anxiety disorders- [30]. However, it should be reminded that in the above mentioned study the group of older people (>60 years) was very small (n = 19).

The exposure to the earthquake also affected the frequency of DNBs. An increased presence of disruptive nocturnal symptoms was observed in the L’Aquila citizens and in the population living within 40 km (Group 2) compared to the other participants. Also in this case, the effect was present partialling out the depression contribution. Interestingly, the regression approach on PSQI-A scores showed that distance and depression have a comparable (in terms of explained variance) influence on this index. It is of note that DNBs are more specifically related to the traumatic experience, and that PSQI-A scores >4 are highly predictive for discriminating between subjects with and without PTSD [26]. These results point out that the effects on DNBs are more specific and strictly related to the traumatic experience.

PSQI-A surprisingly showed that the younger participants report the highest DNB scores. This result was apparently confirmed by the multiple regression outcomes. However, a closer look to Figure 3 (right panel) clearly indicates that the oldest participants showed higher or similar levels of DNBs compared to younger participants in the populations directly hit by the earthquake (Groups 1-POST and 2), while a relative prevalence of DNB in young adults is evident only in those groups farther from the epicenter (100 km and beyond). Therefore, this unexpected effect does not seem imputable to the direct repercussions of the stressful/traumatic experience, casting some doubts on the psychometric properties of the PSQI-A in subjects not directly exposed to a trauma.

Some studies provided evidence that sleep of PTSD patients is more fragmented than normal [31], [32]. For this reasons it has been proposed that PTSD might reflect a breakdown in normal sleep-dependent processing of emotional memories, leading to a dysfunctional consolidation and strengthening of the detailed trauma memory [31], [33]. Given that the presence of sleep problems subsequent to the exposure to traumatic episodes has been linked to an increased likelihood of developing PTSD [27], [33], we can hypothesize that sleep problems may function to maintain posttraumatic stress as opposed to only being secondary to the development of PTSD [34]. Indeed, recent studies have suggested that sleep symptoms like difficulties in falling asleep, frequent awakenings, shorter sleep duration, nightmares and anxiety dreams may be the most significant predictors of PTSD [9]. The cumulative effects of sleep loss and sleep disorders have been associated with a wide range of adverse effects on several cognitive functions. As recently shown, young PTSD survivors of the L'Aquila earthquake exhibited a specific deficit in the formation and consolidation of declarative memory, an effect that was significantly related to the sleep disturbances experienced by those patients [35]. This is also consistent with the finding that individuals with frequent nightmares show an impaired performance at several neuropsychological tasks [36]. Unfortunately, in the present study we did not assess psychological distress and PTSD symptoms. This choice was aimed at restricting the number of questionnaires to fill in, in order to obtain the higher number of participants limiting the drop outs. This limitation of the study does not allow to directly evaluate the relations between traumatic experience, psychological distress and sleep complaints.

In conclusion, this study is the first to show a stable, long-lasting (2 years) effect on subjective sleep quality as a direct consequence of the exposure to a natural catastrophic event as an earthquake. This effect was "distance-dependent", with decreasing sleep quality scores as a function of the proximity to the epicenter. Interestingly, the geographic area affected by clinically significant subjective sleep disturbances (Figure 1, right panel, red-orange areas) is substantially larger than the area hit by the earthquake with the greatest seismic intensity (left panel, red areas). Seismic peak intensity, breaking down monuments and buildings, is usually circumscribed both in time - lasting tens of seconds - and in space. In spite of this, our findings clearly suggest that the psychological effects of the same earthquake may be much more pervasive, lasting for years and involving a much larger population.

As a limitation of the study, a part of the central Italy surrounding the L'Aquila territory was not covered by our sample (see Figure 1, right panel). This area of the country is characterized by the presence of small towns and villages that are difficult to reach. Moreover, it should be borne in mind that central Italy is a peculiar territory, characterized by the presence of the Apennine range. The central part of the Apennines is a largely unstable terrain characterized by extensional tectonics that caused several destructive earthquakes in the last 15 years (e.g., Umbria 1997, magnitude 6.4; Molise 2002, magnitude 6.0). We can not exclude that the relatively high presence of sleep disturbances in the Umbria region (Group 6) may be, at least in part, a result of this. Another potential limitation of the study is the absence of a direct control of other factors that could possibly affect sleep quality (e.g. socio-economic status, health, ambient noise, presence of young children at home), so that we cannot confidently exclude their influence on the results.

Finally, it should be recognized that, notwithstanding the statistical significance of our results, their clinical significance is uncertain, especially in the absence of current measures of PTSD or psychological distress. PSQI scores may be indeed influenced also by chronic diseases, such as dementia, obstructive sleep apnea and depression [28]. From an alternative point of view, one could even consider that the relative preservation of sleep quality may be a marker of resilience in earthquake-exposed samples.

From a clinical point of view, the multiple regression analyses suggest that part of the generic sleep complaints (PSQI) may be mediated by depression, while this is not the case for DNBs, that seem to be more strictly and specifically related to the traumatic experience.

Prospectively, our results suggest the importance of implementing preventive strategies to support sleep quality in the aftermath of a highly stressful event, in particular in the elderly, that seems more vulnerable to sleep disturbances after the exposure to the trauma. This prevention is crucial because sleep disturbances negatively affect the daytime emotional and cognitive functioning [37], [38], may have a reinforcement effect on depressive symptomatology and may be a risk factor for PTSD development and maintaining [9].
